# In Vitro/Ex Vivo Release Study of a Ground Umbilical Cord Matrix Loaded with Dexamethasone

**DOI:** 10.3390/jfb15060157

**Published:** 2024-06-05

**Authors:** Florine Grossetete, Charlotte Garot, Emmanuel Crouzet, Xavier Delavenne, Philippe Gain, Laurence Barnouin, Gilles Thuret

**Affiliations:** 1 TBF—Tissue Bank of France, 6 Rue d’Italie, 69780 Mions, France; florine.grossetete@tbf-lab.com (F.G.);; 2 Laboratory Biology, Engineering and Imaging for Ophthalmology, BiiO, Faculty of Medicine, Health Innovation Campus, 10 Rue de la Marandière, 42270 Saint-Priest en Jarez, France; 3 Pharmacology and Toxicology Unit, University Hospital of Saint Etienne, 42000 Saint-Etienne, France; 4 Ophthalmology Department, University Hospital of Saint Etienne, 42000 Saint-Etienne, France

**Keywords:** human umbilical cord, drug release, dexamethasone, ophthalmological diseases

## Abstract

Eye drops containing steroids and antibiotics are widely used to treat a large range of ocular diseases of the ocular surface. They require frequent instillation or a high dosage, which can affect quality of life. We developed a biomaterial from human umbilical cord that can be loaded with drugs before being placed in the inferior conjunctival fornix. In the present work, this viro-inactivated, freeze-dried, and sterile foam was loaded with dexamethasone phosphate. We studied the release kinetic of 100 mg of biomaterial loaded with 100 µg of dexamethasone phosphate. Assays have shown that the product can be loaded with 100 µg of dexamethasone and allows a progressive release over time for at least 48 h. In addition, when compared with the instillation of the same dexamethasone quantity (100 µg), instilled regularly via eye-drop solution at 0.79 mg/mL, the drug penetration through corneal tissues was better with the dexamethasone-loaded biomaterial.

## 1. Introduction

Topical steroids and antibiotics are widely used in ophthalmology to treat a large range of anterior segment diseases: keratitis, conjunctivitis, uveitis, dry eye, scleritis or episcleritis [1]. It is estimated that 95% of the corticosteroids and antibiotics products used in ophthalmology are solutions. Their manufacturing is easy and mastered by pharmaceutical companies.

Yet, this therapeutic form presents several disadvantages [2]: (1) It is estimated that 95% of the drug is washed away after a few blinks because of lacrimal drainage [1,3]. (2) In some severe diseases such as uveitis, this clearance phenomenon leads to an instillation of eye drops every hour, with a significant impact on the patient’s quality of life [4]. (3) Lacrimal drainage leads the active substances to the nasal mucosa, which is a direct pathway to the systemic circulation [1]. (4) Eye drops must be instilled in the conjunctival cul-de-sac and may require the formation of the patient by the medical staff. Different reports have shown that poor patient compliance with eye drops is a major obstacle to treatment efficiency [5,6].

Dexamethasone is one of the most prescribed steroids eye drops. When administered as a solution, the half-life is estimated between 3 and 6 h [7], explaining the need for frequent instillations. The corneal penetration is poor and low amounts of drug reach the anterior chamber. It requires the instillation of repeated high drug concentrations that can lead to ocular side-effects (increase in intraocular pressure that can lead to a secondary glaucoma) [8].

In this context, research groups have been working to develop different ophthalmic preparations, technologies, or medical devices to carry active substances and to allow a prolonged, homogeneous, and efficient release of the drug through the different eye layers. These products must be sterile, easy to store and to use, and they should not disturb the vision nor cause irritation or foreign body sensation [9].

The literature shows that the most studied technologies are polymer-based delivery plans, mucoadhesive dosage forms, ocular inserts, collagen shields, and drug presoaked hydrogel type contact lens [1,9,10]. Collagen shields often present a quick release of approximately 70% of the drug within the first two hours [11,12] and intraocular injections are invasive with rare but potentially blinding side effects like endophthalmitis [13].

For example, the product Mydriasert^®^, which is an oblong insoluble ophthalmic insert produced and sold by Laboratoire Théa (Clermont-Ferrand, France), contains the equivalent of one drop of mydriatic eye drops. The use of this insert enables stable pupillary dilation, with quantities of active substances 5 to 10 times lower than those administered by eye drops [14].

TBF is a French tissue bank working with human placental tissues. We developed an umbilical cord-based matrix that can be gradually eliminated over time due to its biological composition, and we wanted to evaluate its propensity to be loaded with drugs. The biomaterial can be molded into regular strip that can be inserted and well-tolerated in the inferior conjunctival fornix.

The tissue of interest is the umbilical cord lining. It is known to be rich in collagen, proteoglycans, and glycosaminoglycans such as hyaluronic acid, making it a good candidate for sustained release of various drugs. Hyaluronic acid is a non-sulfated glycosaminoglycan of a high molecular weight that is highly present in the human umbilical cord (4100 µg/mL [15,16]). We supposed that the additional presence of proteoglycans in the human umbilical cord lining could increase the absorption properties of the biomaterial and thus lead to a product with a higher release potential. Indeed, proteoglycans are proteins having a variable number of glycosaminoglycan side chains. Due to their composition, their negative electrical charges and the length of their chain, proteoglycans can bind and trap important amounts of water molecules. It is supposed that they could have the same effect with water soluble drugs [17].

The objectives of this study were to design an ocular insert from human umbilical cord lining and to load it with a defined quantity of dexamethasone, first to evaluate the release kinetics in vitro and then ex vivo on human corneas stored in a bioreactor. Finally, we compared the drug penetration in corneal tissue from the loaded biomaterial versus commercial dexamethasone eye drops.

## 2. Materials and Methods

### 2.1. Biomaterial Preparation

We previously developed an allogenic matrix made of human umbilical cord (hUC) from placental tissues (SygeLIX; Tissue Bank of France (TBF), Mions, France) [18]. Briefly, placental tissues were collected individually after caesarean or vaginal delivery in French Hospitals according to European directive 2006/17/CE [19], after obtention of donor’s consent for tissue donation and associated, legally required, serological analyses (hepatitis B and C, human T-lymphotropic and human immunodeficiency viruses and syphilis). Placental tissues were transported to TBF facilities in 0.9% sodium chloride solution (Versol, Laboratoire Aguettant, Lyon, France) at +4 °C. Then, in a clean room, the hUC was washed with 0.9% NaCl to remove blood clots. After immersion in sterile water (Versol, Laboratoire Aguettant, Lyon, France) between 2 and 72 h at +4 °C, the hUC was opened and the vessels were manually removed from the lining. The hUC linings (hUCL) were then stored in dry conditions at −80 °C until use for up to 24 months without biological changes.

Umbilical cord linings were then virally inactivated according to a chemical treatment consisting of a succession of 70% ethanol (Chimie+, Saint-Paul-de-Varax, France) and hydrogen peroxide (Chimie+) baths. This treatment did not alter the proteins constituting Wharton’s jelly. The tissues were then grounded with a ball mixer mill into a gel form that could be molded into different forms, thanks to its viscous properties. The different forms were then dehydrated by freeze–drying technology, packaged in Tyvek/Polyethylene (PET)/polyolefin (PE) bags, SÜDPACK MEDICA, Mareuil-lès-Meaux, France), and sterilized by gamma irradiation (25–32 kGy), allowing its storage for up to 5 years at room temperature (RT). A molded flexible form with a foamy appearance presenting a cohesive texture and regaining volume when exposed to water or other hydrophilic solutions was thus obtained. Its biocompatibility was validated according to ISO 10993 standard [20].

We patented the complete transformation process from umbilical cord lining to the loading biomaterial [18].

### 2.2. Characterization of the Biomaterial

#### 2.2.1. Macroscopic and Microscopic Structure

The biomaterial was rehydrated in phosphate-buffered saline (PBS), then stored in a fixative solution of alcohol/formaldehyde and acetic acid (AFA). Then, samples were dehydrated by successive baths of ethanol, acetone, and xylene, and included in paraffin. Cuts of 5 μm were performed with a microtome and set on nontreated slides.

Immunohistochemistry (IHC): After deparaffinization, antigenic sites were unmasked by treatment with 0.5% hyaluronidase (1 h at RT). Sections were incubated overnight at 4 °C with primary antibodies: human α collagen I—1:2000 (ref. 20111, Novotec, Bron, France); human α elastin—1:2000 (ref. 25011, Novotec, Bron, France); biotinylated HABP—1:1000 (ref. AMS.HKD.BC41, Novotec, Bron, France), in 3% PBS-BSA buffer. After inhibition of endogenous peroxidases by hydrogen peroxide, sections were incubated with the peroxidase-coupled secondary antibody (Envision rabbit, ref. K4002, Dako, Santa Clara, CA, USA). Reaction with its substrate, diaminobenzidine (DAB) (Dako, K3468), reveals antigen–antibody complexes by the appearance of a brown label. Sections were counterstained with Mayer’s hematoxylin, then mounted between slide and coverslip in aqueous medium. The primary antibody was replaced by PBS-BSA 3% as a negative control.

Immunofluorescence (IF): After deparaffinization, “negative control” sections were incubated by treatment with 0.5% hyaluronidase (1.5 h at 37 °C). All sections were incubated overnight at 4 °C with biotinylated HABP diluted 1:100 in 3% PBS-BSA buffer, then 1 h at RT with Streptavidin coupled to Alexa Fluor^®^ 488 (ref. S32354, Molecular Probes, Eugene, OR, USA). Sections were rinsed in a PBS bath containing a few drops of Evans Blue to mask non-specific fluorescence. Sections were then mounted between slide and coverslip with mounting medium containing DAPI (Slow fadeTM Gold antifade reagent with DAPI, ref. S36938, Invitrogen, Waltham, MA, USA).

#### 2.2.2. Protein Composition

The biomaterial was homogenized in 10 mL of TRIZMA base 25 mM, pH 8.0. A total of 100 mg of homogenate was solubilized, reduced, and alkylated by boiling and sonication in iST LYSE buffer (Deoxycholate, TCEP and Chloroacetamide), and the protein concentration was determined by the BiCinchoninic acid Assay (BCA) method. Peptide extracts were prepared according to the iST method (in Stage Tips). A weight of 130 μg of proteins were diluted and digested by LysC trypsin Mix (ThermoScientific Waltham, MA, USA). Peptides were purified by a mixed-mode reverse phase cation exchanger SPE (PreOmics GmbH, Planegg, Germany), dried, and solubilized in 100 μL of 3% acetonitrile, and 0.1% formic acid aqueous solution (ThermoScientific, Waltham, MA, USA). The peptide concentration was determined using BCA method.

A weight of 250 ng of peptides were injected into simplicate for sample. Chromatography was performed using Ultimate 3000 (Dionex, Sunnyvale, CA, USA) equipped with C18 (75 μm × 50 cm, 2 μm material) column applying a 2.5 to 35% acetonitrile gradient at a flow rate of 300 nL/min for 100 min after a 3 min trapping step on a precolumn.

Data were acquired using a Q-Exactive (ThermoScientific, Waltham, MA, USA) mass spectrometer. Mass spectrometry scan was performed with a resolution of 70,000 and an accumulation time of 60 ms. MS/MS scan was performed with a resolution of 17,500 on the 10 most intense ions of each cycle with an accumulation time of 60 ms. A total of 5714 cycles were performed, thus an average of 17 cycles per chromatographic peak.

Proteins were identified using SEQUEST-HT algorithm against a database gathering Homo sapiens proteome, mined from UniProt KnowledgeBase [21].

### 2.3. Loading and Extraction In Vitro

For this assay, biomaterials were prepared as discs of 1 cm diameter. Dexamethasone release kinetics was studied as follows. Three biomaterials were preloaded with 200 μL of dexamethasone solution at 1 mg/mL (DEXAFREE 1 mg/mL, Laboratoires Théa Pharma, Clermont-Ferrand, France), and dried in the dark at RT under laminar flow for 30 min. Preloaded biomaterials were then immersed in 2 mL of 0.9% NaCl solution (Versol, Laboratoire Aguettant, Lyon, France), to simulate lacrimal liquid and placed under smooth mechanical agitation at RT. Extraction timepoints were planned at 30 min, 60 min, 90 min, 4 h, 15 h, 24 h, and 48 h. At each sampling time point, the 2 mL extraction medium was removed and transferred into a cryotube stored at +4 °C until the end of the experiment. Then, the 2 mL were replaced by fresh 0.9% NaCl solution. Finally, the dexamethasone quantity in liquid and biomaterial samples for each point was measured using an ELISA dosage kit: DEX (Dexamethasone) ELISA Kit supplied by Elabscience (Houston, TX, USA). The kit is based on DEX antibody-DEX antigen interactions (immunosorbency) and an HRP colorimetric detection system to detect DEX antigen targets in samples. The biomaterial and the liquid extracts were prepared according to the supplier protocol provided in the ELISA kit. Briefly, biomaterial samples were pretreated according to the protocol “Pretreatment of tissue”, whereas the liquid samples were prepared according to the protocol “Pretreatment of milk samples”. The optical density measurement was performed with a Tecan SUNRISE^®^ microplate reader (Männedorf, Switzerland).

### 2.4. Preclinical Assay in a Corneal Bioreactor

#### 2.4.1. Cornea Selection and Storage in a Bioreactor

Three pairs of corneas from the Besançon eye bank, unsuitable for transplantation, with an endothelial cell density (ECD) greater than 1000 cells/mm^2^ and with an organ culture storage time of less than 42 days (6 weeks) prior to bioreactor insertion were used. The corneal bioreactor was developed to recreate a more physiologic environment to the ex vivo cornea. It restored a pressure in the endothelial chamber equivalent to intraocular pressure (21 mmHg), while continuously renewing the medium in the epithelial and endothelial chambers. These parameters played important roles in corneal homeostatis. We previously showed that storage into the bioreactor (also called active storage machine, by assimilation to machine perfusion used for vascularized organs) significantly improved corneal cells survival compared to conventional—passive storage in the same medium [22,23]. In addition, the corneas were placed in the bioreactor 14 days before the start of the study, to obtain corneas with an epithelium close to the physiological state as previously shown [24]. The medium used in the 2 chambers was CorneaMax (Eurobio Scientific, les Ulis, France), with a flow rate of 2.6 μL/min and 21 mmHg of intra ocular pressure. The bioreactor was kept at 31 °C in a dry incubator with 5% CO_2_.

#### 2.4.2. Condition of Corneal Exposure to Dexamethasone Phosphate

Biomaterials were prepared as small cylinders of 10 mm diameter and 3 mm height to fit in the epithelial chamber of the bioreactor. Two solutions of dexamethasone at 1 mg/mL and 0.79 mg/mL were prepared from a powder of Dexamethasone—Water Soluble (D2915, Sigma-Aldrich, St. Louis, MO, USA) diluted in Buffer Salt Solution (BSS). It was planned to load the biomaterial with 100 µg of dexamethasone. The 0.79 mg/mL solution was prepared for the eye-drop group as the aim was to provide the same dexamethasone quantity but divided into 3 drops instilled at different time points.

For each pair of corneas, a biomaterial preloaded with 100 μL of dexamethasone at 1 mg/mL was deposited on the corneal dome of the right eye. The left cornea was treated with drops of 38 μL (the average volume of eye drops) of dexamethasone at 0.79 mg/mL according to the following time points:

- Pair 1: Left cornea treated 1 time with dexamethasone at T_0_.
- Pair 2: Left cornea treated 2 times with dexamethasone at T_0_ and T_5h45_.
- Pair 3: Left cornea treated 3 times with dexamethasone at T_0_, T_5h45_ and T_11h45_.

These time points were chosen to correspond to the minimal bioavailability of dexamethasone for the cornea treated with eye drops.

Each drop was deposited on the corneal apex at the timings given above. Immediately after biomaterial placement or drop instillation, the bioreactor was returned to its mount with the epithelial chamber moistened by a meniscus of BSS.

At the end of the experiment, corneas were dissected as follows to analyze each layer separately: the epithelium was scraped with a surgical spatula, and the stroma + its endothelium was separated from the sclera with scissors. The endothelial chamber media was harvested, as well as the three biomaterials. The positive control consisted of a biomaterial loaded with 100 μL of dexamethasone at 1 mg/mL. All samples were weighed, then frozen at −80 °C. The theoretical dexamethasone stock solution at 1.00 mg/mL and 1 drop of 38 μL at the 0.79 mg/mL solution were also frozen for quantification.

#### 2.4.3. Dexamethasone Extraction and Quantification

The extraction and quantification methods have been previously validated for this specific study.

Each tissue (epithelium/stroma + endothelium) recovered at the end of the study was ground in 1 mL of sterile water for homogenization using a manual tissue grinder (ref.065359, Dutscher, Bernolsheim, France). An initial centrifugation was performed at 4700 rpm for 3 min to sediment the bulk of the solid tissue. The supernatant was recovered and centrifuged again at 4000 rpm for 10 min to remove the last traces of solids. The supernatant was recovered for quantification.

The quantification was performed by HPLC-MS/MS, with an HPLC column BEH C18 2.1 mm × 50 mm (Waters, Milford, MA, USA) using phase A: 100% water + 0.1% formic acid and phase B: 100% acetonitrile + 0.1% formic acid. The retention time was 1.41 min.

The mass spectrometry was performed with QExactive Plus system (ThermoFisher, Waltham, MA, USA) on tSIM mode.

All sample tubes were numbered and anonymized for blind quantification.

The theoretical dexamethasone concentration of the positive control biomaterial was calculated, as described in Formula (1):

(1) $${\left[Dexamethasone\right]}_{theorical;biomatT+}=\frac{{\left[Dexamethasone\right]}_{measured;initial.sol}\times {VolumeLoaded}_{initial.sol}}{{Weight}_{biomat\;T+}}$$

- «[Dexamethasone]_measured;initial.sol_» represents the concentration of dexamethasone that has been measured in the initial solution in mg/mL;
- «VolumeLoaded_initial.sol_» represents the volume of dexamethasone initial solution, loaded on the biomaterial in mL;
- «Weight _biomat T+_» represents the weight of the biomaterial before quantification in mg.

For the liquids, dexamethasone quantity was measured in mg/mL.

For the tissues, it was converted in mg/g of tissue, according to Formula (2):

(2) $${\left[Dexamethasone\right]}_{tissue}=\frac{{\left[Dexamethasone\right]}_{liquid.extract}\times {{Volume}_{extraction}}_{\;}}{{Weight}_{tissue}}$$

- «Volume_extraction_» represents the volume used to grind the tissues (here 1 mL);
- «[Dexamethasone]_liquid.extract_» is the dexamethasone concentration measured by HPLC in the liquid extract in mg/mL;
- «Weight_tissue_» represents the weight of the tissue evaluated before quantification in mg.

Relative differences between theoretical concentration and measured concentration were calculated according to Formula (3):

(3) $$Relative\;difference=\frac{{\left[Dexamethasone\right]}_{measured}\times {{\left[Dexamethasone\right]}_{theoretical}}_{\;}}{{\left[Dexamethasone\right]}_{theoretical}}$$

It should be noted that this method was previously validated for the dexamethasone quantification in these specific conditions.

### 2.5. Statistical Analysis

The data were reported as mean ± standard deviation (unless otherwise indicated). GraphPad Prism 9.5.1 for Mac software (GraphPad Software, San Diego, CA, USA) was used for statistical analysis. For all statistical analyses, differences were considered significant at *p* < 0.05. The differences in mean drug amount in total corneal tissues between biomaterial group and eyedrop group were tested by an unpaired *t*-test.

## 3. Results

### 3.1. Biomaterial Characteristics

**Figure 1:**
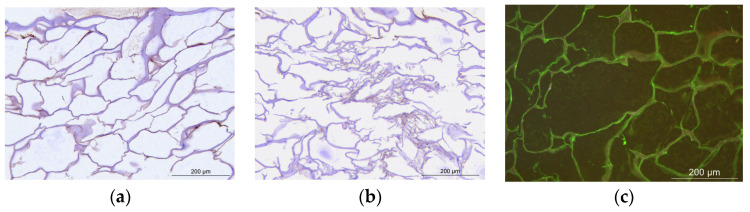
Microscopic observation of the biomaterial structure after immunohistological analysis. (**a**) collagen, (**b**) elastin, and (**c**) hyaluronic acid.

The biomaterial consisted of a dehydrated white soft foam. After being compressed, it could return to its original shape. Protein identification using LC-MS/MS method combined with ELISA kit dosage for detection showed that it contained growth factors such as TGF-β1 (0.216 μg per mg of biomaterial), glycosaminoglycans: hyaluronic acid (2 mg/mL of Wharton jelly), and glycoproteins: collagen and elastin (observed in the structure by IHC staining and presented in Figure 1).

As it was also observed on the IHC staining images (Figure 1), the biomaterial has an airy structure, the fibers were organized into an alveolus gelatinous structure and new bonds were formed between the fibers.

Due to its structure and composition, the biomaterial was absorbent. It was rehydrated with different types of liquids, such as 0.9% NaCl or more viscous such as blood. The presence of proteoglycans in its composition was responsible for its hydrophilic properties.

### 3.2. Loading and Extraction In Vitro

For biomaterials loaded with 200 μg of dexamethasone, the release profile showed an initial progressive release of the dexamethasone for the first 6 h of experiment. Then, a plateau was reached, and lower quantities of drugs were released until 45 h. After 48 h of extraction, 54.5 ± 4.5% of the drug have been released. At the end of the experiment, the dexamethasone quantity remaining in the biomaterial was evaluated at around 53.12 μg.

### 3.3. Dexamethasone Release

Dexamethasone concentrations were measured at 0.990 mg/mL (−1.01% relative difference from the theoretical concentration) and 0.810 mg/mL (+2.50% relative difference from the theoretical concentration), respectively, for the stock solution and the eye drop solution (Table 1).

**Figure 2:**
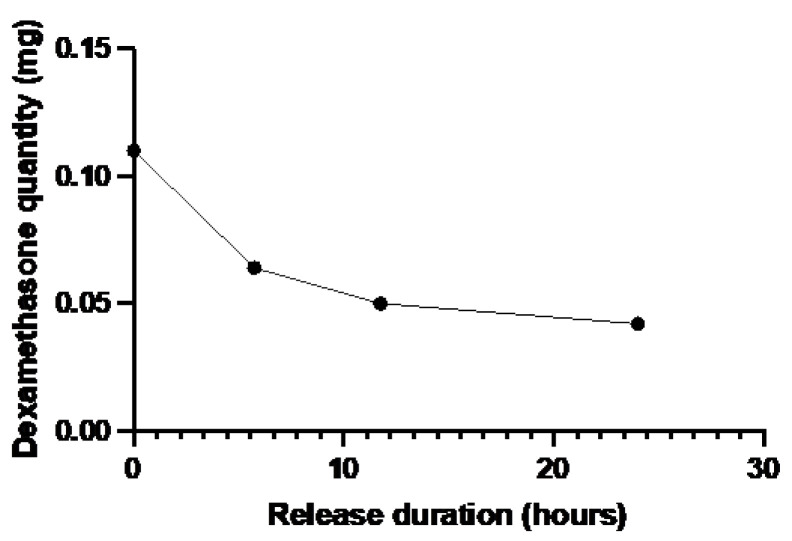
Residual dexamethasone quantity in the biomaterial over extraction time.

The biomaterial weight ranged from 65 to 122 mg. In view of these weight differences, it was more relevant to consider the total amount of dexamethasone present in the biomaterial, rather than the mass concentration (mg/g tissue). The positive control, equivalent to T_0_ in the study, was loaded with 0.110 mg of dexamethasone. In the first 6 h, the biomaterial discharged rapidly, reaching a total quantity of 0.064 mg of dexamethasone after 5 h 45 min of contact with the cornea. After a contact of 11 h 45 min between the loaded biomaterial and the cornea in the bioreactor, drug release slowed down, with a total of 0.050 mg at T_11h45_ and 0.042 mg at T_24h00_. All results were detailed in Table 2 below and illustrated on Figure 2.

### 3.4. Dexamethasone Distribution in Ocular Tissues

As presented in Table 3, the epithelium of corneas receiving the dexamethasone-loaded biomaterial had drug concentrations of 0.137, 0.312, and 0.133 mg/g, respectively for pairs 1, 2, and 3. These concentrations were always higher than those of the corneas receiving the drops, which had drug concentrations of 0.035, 0.050, and 0.019 mg/g, respectively, for pairs 1, 2, and 3.

As in the epithelium, dexamethasone concentrations in stroma and endothelium of corneas from the biomaterial group were higher than in stroma and endothelium of corneas from the drop group.

**Figure 3:**
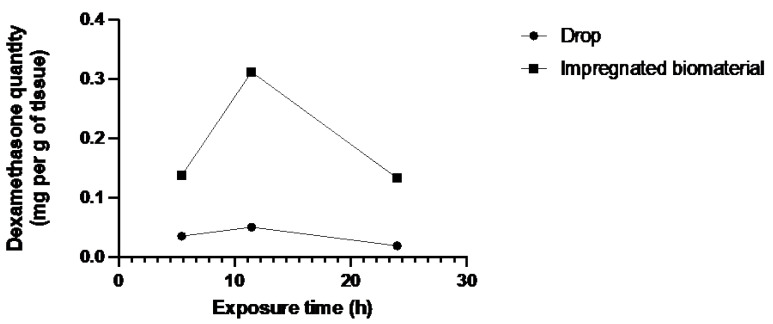
Dexamethasone quantity in the epithelium of both groups over exposure time.

**Figure 4:**
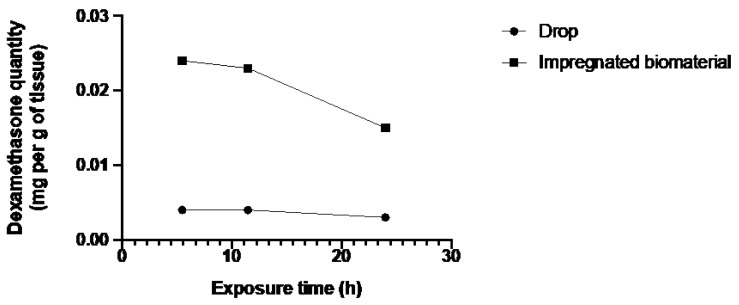
Dexamethasone quantity in stroma and endothelium of both groups over exposure time.

**Figure 5:**
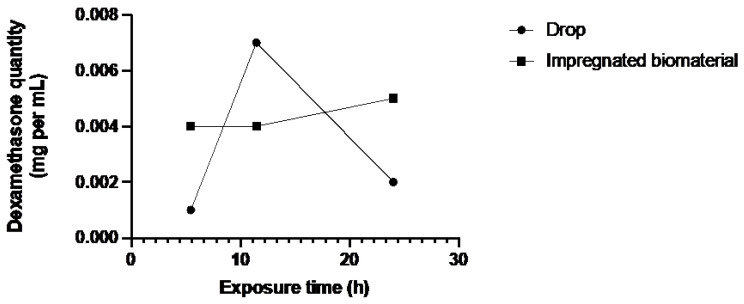
Dexamethasone quantity in endothelial chamber medium of both groups over exposure time.

Figure 3, Figure 4 and Figure 5 showed a higher amount of drug in the different parts of the corneal tissues for the biomaterial group. The additional instillation of eye drops in the second (11 h 45 min) and third (24 h) groups did not lead to an increase in the dexamethasone quantity in the different corneal layers.

Also, the total amount of drug in corneal tissues (Epithelium + Stroma + Endothelium) was significantly higher at each time point in the biomaterial group than in the drop group.

## 4. Discussion

The in vitro results demonstrated the potential of the biomaterial for the progressive release of dexamethasone into water, simulating tears fluid at the ocular surface. Also, the biomaterial remained intact and still contained dexamethasone even after 48 h of extraction, suggesting its capability to facilitate progressive release under physiological conditions. It is also likely that, in vivo in the presence of lacrimal enzymes [25], the biomaterial may exhibit signs of degradation after 48 h, thereby enhancing drug release over time. This sustained release capacity suggests that the biomaterial could offer a promising solution for drug delivery under physiological conditions akin to those encountered at the eye surface.

Furthermore, the versality of the biomaterial, which can be molded into various forms, suggests its potential for diverse applications based on the defect filling principle.

Additionally, exploring the loading capacity of the biomaterial with different substances and assessing their release kinetics would be interesting for further research topics.

**Figure 6:**
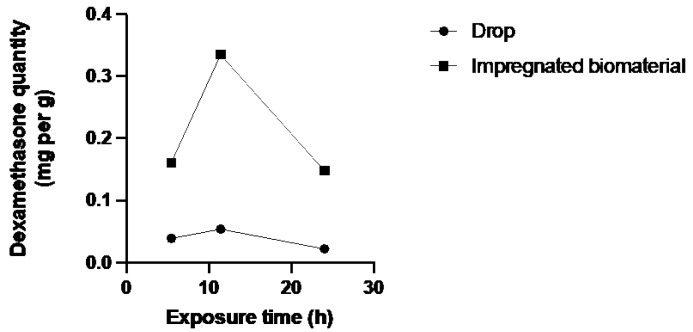
Dexamethasone total quantity in all corneal tissues for both groups over exposure time.

The bioreactor assay was performed on human corneas, which is a considerable strength of this study. In all three tissues, there is a notable trend indicating superior absorption of dexamethasone by the biomaterial group. Figure 3 and Figure 5 depict a dosing point at 11 h 45 min where the biomaterial group (Figure 3) and the eye drop group (Figure 5) exhibit higher concentrations than the overall trend. This deviation from the general trend could be attributed to two potential factors: either an operational issue occurred during dosing, or dexamethasone diffusion through the tissue in the bioreactor differed due to the non-vascularized nature of the stroma, resulting in tissue-specific diffusion patterns for each cornea. Despite these localized variations, when evaluating the overall trend across the entire study duration, as depicted in Figure 6, a consistent pattern emerges. Specifically, tissues from the biomaterial group exhibit higher concentrations of dexamethasone compared to those from the eye drop group. Remarkably, this difference persists despite both groups receiving an equivalent total amount of drug over the study.

This confirms that the biomaterial allows progressive release of the drug during a prolonged period of contact. Regarding the dexamethasone quantity in the different corneal layers, the corneas treated with the biomaterial showed a significantly better penetration of the drug into the different layers compared to the eye drops group despite the re-instillation of drops during the experiment. However, it must be considered that for this bioreactor assay, the biomaterial was in contact with the cornea, whereas for in vivo use, the strip is inserted in the inferior conjunctival fornix. In addition, we measured the average quantity in each layer of tissue, so we cannot conclude whether there are local variations depending on proximity to the biomaterial. It would be interesting to check in vivo if the ocular tissue directly in contact with the biomaterial has the same dexamethasone concentration than tissues localized at a distance. Another limitation is the limited number of human corneas available for scientific use. This is counterbalanced by the quality of the tissue rehabilitated in the bioreactor.

Furthermore, the results obtained for the eye drop group in the bioreactor must be considered carefully as the system is a closed chamber. Consequently, the concentrations measured were likely maximized as there was no tear clearance. In vivo, the result for the eye drops group would most probably be inferior to the quantity observed in vitro in the bioreactor.

Given the promising results, the next validation steps involve animal experimentation followed by phase 1 clinical trial. These steps will facilitate real-time efficacy assessments, determination of the optimal drug loading quantity on the biomaterial and evaluation of local systemic toxicity. Additionally, they will provide insights into the biomaterial’s lifespan in physiological conditions.

## 5. Conclusions

The matrix from human umbilical cord can be loaded with dexamethasone phosphate and allow its gradual release. This innovative approach offers several advantages over traditional eye drops used in treatment of ocular disorders. First, it eliminates the need for repeated instillation, offering a more convenient solution for patient and reducing the treatment burden. Also, the matrix enables prolonged release of the drug, which enable higher concentration to be achieved locally, improving treatment efficacy. By prolonging the contact time between the eye and the active substance, this approach could potentially improve clinical outcomes and reduce complications associated with ocular treatment.

Further research is needed to optimize this technology and explore its potential in other areas of ophthalmology, and we could study the efficacy of this matrix with other drugs.

This technology could pave the way for new advances in the treatment of eye diseases and improve the quality of life of patients suffering from these pathologies.

## 6. Patents

Barnouin, L (2022): Composition comprising Wharton’s jelly, method for preparing same and uses thereof, Patent WO2019038411A1. World Intellectual Property Organization [18].

Gain et al. (2016): “Medical device intended for the long-term storage of a cornea, or for ex vivo experimentation on a human or animal cornea”. US20160029618A1 [26].

## Figures and Tables

**Table 1:** Dexamethasone concentration of the tested solutions.

Solution	Theorical Concentration	Measured Concentration	Relative Difference
Dexafree collyrium	1.000 mg/mL	0.990 mg/mL	−1.01%
38 μL of prepared eye-drop solution	0.790 mg/mL	0.810 mg/mL	+2.50%

**Table 2:** Dexamethasone quantity and concentration in the biomaterial.

	Total Quantity (mg)	Biomaterial Weight (g)	Concentration (mg/g Tissue)
Biomaterial—positive control (H0)	0.110	0.111	0.993
Biomaterial—Pair 1 (H = 5 h 45 min)	0.064	0.098	0.656
Biomaterial—Pair 2 (H = 11 h 45 min)	0.050	0.122	0.411
Biomaterial—Pair 3 (H = 24 h 00 min)	0.042	0.065	0.649

**Table 3:** Dexamethasone quantity in each ocular tissue.

	Dexamethasone Quantity in Epithelium (mg per g of Tissue)		Dexamethasone Quantity in Stroma and Endothelium (mg per g of Tissue)		Dexamethasone Quantity in Endothelial Chamber Medium (mg per mL)	
Time	Loaded biomaterial	Drop	Loaded biomaterial	Drop	Loaded biomaterial	Drop
5 h and 45 min	0.137	0.035	0.024	0.004	0.004	0.001
11 h and 45 min	0.312	0.05	0.023	0.004	0.004	0.007
24 h	0.133	0.019	0.015	0.003	0.005	0.002

## Data Availability

The datasets used and/or analyzed during the current study are available from the corresponding author on reasonable request.
